# Coevolutionary training of phages can be more successful in several small, relative to single large, habitats

**DOI:** 10.1002/mlf2.12158

**Published:** 2025-03-12

**Authors:** Xiao Liu, Quan‐Guo Zhang

**Affiliations:** ^1^ State Key Laboratory of Earth Surface Processes and Resource Ecology and MOE Key Laboratory for Biodiversity Science and Ecological Engineering, College of Life Sciences, Beijing Normal University Beijing China

## Abstract

Evolutionary training of phages can help to counter bacterial resistance evolution. Here, we address whether metapopulation processes can enhance the evolution of phage infectivity. Our experiment with a model bacterium‐phage system supported a prediction of long‐term fluctuating selection dynamics. Specifically, metapopulations of several small habitats showed greater total infectivity ranges by supporting more diverse phages, compared with single large populations. Crucially, the advantage of several small habitats was conditioned on blocking bacterial dispersal within metapopulations. We conclude that well‐designed metapopulation training programs can be useful for quick and easy preparation of cocktail phage materials.

The use of phages to treat harmful bacteria and prevent infections in clinical or agricultural contexts is now attracting resurgent interest^1^, ^2^. However, bacteria can readily evolve resistance to phages. Obtaining phage materials that slow or prevent bacterial resistance evolution is crucial for the success of phage applications. Evolutionary training of phages on target hosts in laboratory environments before using them may enhance their controlling effects, or even prevent bacterial resistance evolution^3^, ^4^. However, improved phage infectivity should not be taken for granted in phage training programs. Phages typically have lower evolutionary potential compared with host bacteria during coevolution. Changes in bacterial resistance can result from a number of modifications including alteration or loss of phage receptors, but the evolution of infectivity of phages usually requires very specific changes including those depending on stepwise acquisition of multiple mutations^5^. As a result, phages may lag behind host bacteria in coevolution and even go extinct^6^, ^7^. It is therefore crucial for phage training programs to mitigate the evolutionary disadvantage of phages relative to host bacteria.

A number of ecological conditions may alter the evolutionary potentials of bacteria and phages. For example, phages may evolve greater infectivity and escape the fate of extinction in larger habitats. This is because increasing population sizes show a diminished enhancement effect on evolutionary speed^7^; thus increasing habitat sizes would benefit both bacteria and phages, but with a greater effect on the latter which usually have lower evolutionary potential. Greater rates of gene flow into phage populations may also promote their local adaptation^8^, ^9^.

Here, we address whether metapopulation processes promote phage infectivity evolution. Specifically, we studied single large (SL) or several small (SS) habitats with the same total habitat size (SLOSS). A phage preparation may show greater infectivity if it contains multiple specialist genotypes with complementary infectivity profiles, or particular generalist genotypes with broader infectivity ranges. These two scenarios may be achieved under different modes of coevolution. Fluctuating selection dynamics are characterized by frequency oscillation of specialist bacterial and phage genotypes^10^. SS habitats may allow for greater genotype diversity due to population divergence among local habitats, and phages from SS habitats are predicted to have greater total infectivity ranges compared with SL habitats (Figure S1). Under arms race‐like coevolution, recurrent selective sweeps take place favoring generalist bacterial genotypes with broader resistance mechanisms and generalist phage genotypes with broader infectivity ranges. Evolutionary speed is expected to be faster in SL habitats than in SS habitats, with more rapid emergence of generalist phage genotypes (Figure S1). This is because habitat fragmentation reduces effective population size, with slower rates of beneficial mutation fixation and larger chances of deleterious mutation accumulation^11^, ^12^.

We carried out a SLOSS coevolution experiment using a model system. The bacterium *Pseudomonas fluorescens* SBW25 and its lytic phage SBW25Φ2 typically undergo arms race‐like changes during short periods of coevolution^13^, ^14^. However, a transition to fluctuating selection dynamics may take place in the long run, and fitness costs associated with resistance and infectivity traits may contribute to the termination of arms race‐like dynamics^15^, ^16^. In our experiment, the bacterium and the phage coevolved for 16 cycles of population propagation (transfers). Bacterial/phage populations evolved either as single cultures in SL habitats, or as metapopulations each of which consisted of five SS habitats. Dispersal among local populations within a metapopulation may involve both bacteria and phages, or only phages (Figures 1A and S2). There was no extinction of bacteria throughout the experiment. Phage populations persisted until the end of the experiment in all evolution lines except for one SL population and one SS metapopulation with bacteria/phage dispersal. Phage infectivity was measured using common pools of bacterial isolates as the challenge targets (Figure S2). For each SL population, a single measure of infectivity was estimated. For each SS metapopulation, infectivity of the metapopulation (mixed sample of five local populations), as well as every local population, were measured. The difference between metapopulation‐level infectivity and the maximum local population‐level infectivity was termed an “over‐infectivity” index (Figure S2), and positive values of this index indicate complementarity in infectivity profiles among the local populations.

**Figure 1:**
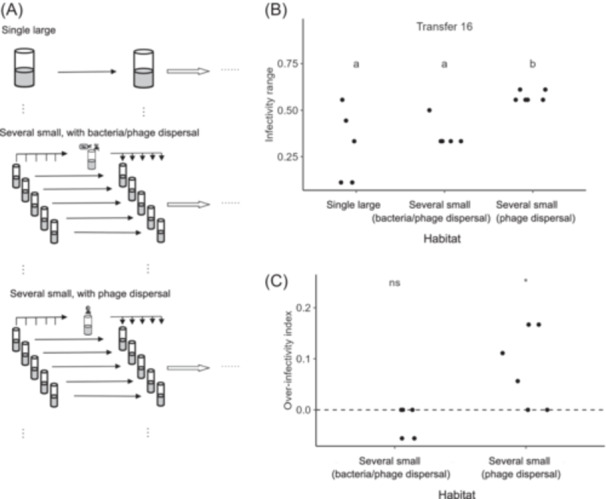
Experimental design and results. (A) A graphical illustration of the experimental design. (B) Infectivity ranges of phage populations, where infectivity of a metapopulation was measured based on the mixed phage sample from its five local populations. Habitats annotated with a same lowercase letter showed no significant difference (based on two‐sample Wilcoxon tests, *p*_adj_ > 0.05; see details in Table S2). (C) Over‐infectivity of metapopulations, calculated as the difference in infectivity between a metapopulation (mixed sample of local populations) and the particular local population with the greatest infectivity. The difference of mean values from the expected value of zero was indicated by annotated symbols, with asterisk and “ns” indicating significant and nonsignificant differences, respectively (based on one‐sample *t* test or one‐sample Wilcoxon test; see details in Table S3). Scatter points were jittered along the horizontal direction to avoid overlapping.

The ancestral phage strain showed an infectivity score of zero against the reference bacterial pools, and the infectivity of evolved phages was significantly greater than the ancestral phage (Table S1). Though no impact of metapopulation processes on phage infectivity evolution was found at an early point in time (transfer 4; Figure S3; Tables S2 and S3), significant differences between evolution lines were observed at the end of the experiment (transfer 16). SS metapopulations with phage‐only dispersal had greater infectivity than the other two types of evolution lines (Figure 1B and Table S2). Metapopulation‐level infectivity was greater than the maximum local population infectivity in SS metapopulations with phage‐only dispersal, but not in those with bacteria/phage dispersal (Figure 1C and Table S3).

Results from the end of the experiment (transfer 16) were consistent with a prediction based on fluctuating selection dynamics of coevolution. Specifically, SS metapopulations with phage‐only dispersal had positive over‐infectivity scores, that is, their metapopulation‐level infectivity was greater than the maximum local population infectivity (Figure 1C). Thus, phages in different habitat patches must have had complementary infectivity profiles. The SS metapopulation with phage‐only dispersal had greater metapopulation‐level infectivity than SL populations (Figure 1B). However, when both bacteria and phages migrated among local habitats within metapopulations, the phages did not evolve greater infectivity ranges. These findings imply that coevolutionary trajectories diverged among local habitats in SS metapopulations in the late stage of the experiment, and that the heterogeneity among habitat patches in coevolutionary dynamics may only be maintained in the absence of host dispersal. Previous research suggested that increased dispersal rate of phages, relative to bacteria, can enhance phage local adaptation to host bacteria, which the authors attributed to increased genetic variation in local phage populations^8^, ^9^. Our results here suggest that maintaining the heterogeneity in host population composition among habitats may be crucial for promoting phage genetic diversity at the metapopulation level.

Our observations at transfer 4 (Figure S3) did not support the prediction that phages can evolve greater infectivity in SL habitats than in SS habitats in the early stage of coevolutionary training due to greater evolutionary speed (Figure S1). One possible explanation is that local populations in an SS metapopulation showed limited divergence in genotype composition during this very early stage of coevolution and our experimental dispersal (approximately 0.8% immigration from other patches within the metapopulation) has been high enough to homogenize the metapopulation.

Cocktail therapy is the routine practice in phage applications, the success of which depends on collecting phage strains with complementary infectivity profiles^17^, ^18^. The present study suggests that well‐designed metapopulation training programs may obtain desirable phage materials without laborious identification and trait profiling work. Our results call for future studies to compare phage materials from metapopulation training experiments with well‐developed cocktail preparations using study systems of clinical or agricultural importance. Evolutionary training approaches developed in phage training programs may also be extended to biocontrol practices for other types of harmful organisms including insect pests and weeds.

## AUTHOR CONTRIBUTIONS

**Xiao Liu**: Data curation (equal); formal analysis (equal); investigation (equal); writing—original draft (equal). **Quan‐Guo Zhang**: Conceptualization (equal); formal analysis (equal); funding acquisition (equal); investigation (equal); methodology (equal); project administration (equal); writing—original draft (equal).

## ETHICS STATEMENT

This work did not require ethical approval from a human subject or animal welfare committee.

## CONFLICT OF INTERESTS

The authors declare no conflict of interests.

## Supporting information

Supporting information.

## Data Availability

Data associated with this study will be available at https://figshare.com/s/bf79cb30a11be2e51a48.
