# Supplementary material for: Coevolutionary training of phages can be more successful in several small, relative to single large, habitats
Source: mLife. 2025 Mar 12;4(2):223–5. doi: 10.1002/mlf2.12158 (PMC12042113; doi:10.1002/mlf2.12158)
Supplement: Supplementary file 1 — Supporting information. [file MLF2-4-223-s001.docx]

**Supplementary Information**

**Coevolutionary training of phages can be more successful in several small, relative to single large, habitats**

**Xiao Liu and Quan-Guo Zhang**

**Text S1 Supplementary methods**

**Strains and culture conditions**

The bacterium *Pseudomonas fluorescens* SBW25 [^1^](#_ENREF_1), and its lytic bacteriophage virus SBW25Φ2 [^2^](#_ENREF_2) were used in the present study. Cultures were grown at 26°C in static microcosms, centrifuge tubes with lossened lids containing LB medium (10 g L^-1^ tryptone, 5 g L^-1^ yeast extract, and 10 g L^-1^ NaCl). Samples of cultures were frozen stored at -80°C with glycerol (40% glycerol).

Two types of U-bottom centrifuge tubes were used to establish experimental microcosms (Fig. S4; Hengchao Limited, Nantong, China). The inner diameters of the 10- and 100-mL tubes were 16 and 37 mm, respectively. When the small and large tubes were loaded with 2 and 10 mL of liquid medium, respectively, the depth of medium was ~20 mm in both tubes.

Isolation of phages from cultures involved mixing 0.4 mL of culture and 40 μL of chloroform, lysing bacterial cells by vortex, and centrifuging cultures at 15,000 g for 2 min to pellet the bacteria debris. A suspension of phage particles would be left in the supernatant.

**The SLOSS coevolution experiment**

Figure S2 illustrated the design of our experiment. Bacterial and phage populations coevolved in either single large habitats (SL), or metapopulations of several small habitats (SS). One SL habitat consisted of one 100-mL centrifuge tube containing 10 mL of liquid medium; and one SS metapopulation consisted of five 10-mL tubes each of which contained 2 mL of medium. A total of three types of evolution lines were set up: SL populations, SS metapopulations with bacteria/phage dispersal, and SS with phage-only dispersal. There were six replicates for each type of evolution lines.

Every microcosm was initially inoculated with approximately 10^7^ mL^-1^ of bacterial cells and 10^4^ mL^-1^ of phage particles. Cultures were then propagated for 16 transfers, one transfer every two days. At each transfer, 1% of cultures were added to fresh media. Specifically, 100 μL of culture from each SL population was transferred to 9.9 mL of fresh medium. For an SS metapopulation with bacteria/phage dispersal, a mixed sample was created by drawing 10 μL of culture from each local population. Every new microcosm containing 1.98 mL of fresh medium was inoculated with 19.8 μL of culture from an old microcosm, together with 0.2 μL of the mixed sample. For an SS metapopulation with phage-only dispersal, phages from every local population were used to assemble a pool, 10 μL of phage extract from each local population. Every new microcosm would receive 19.8 μL of culture from an old microcosm, together with 0.2 μL of the mixed phage sample (Fig. S2). The dilution rates for phages were 0.01 in all the three types of evolution lines. The dilution rates for bacteria were 0.01 in SL populations and SS metapopulation with bacteria/phage dispersal, and 0.0099 in SS metapopulations with phage-only dispersal.

**Measurement of phage infectivity**

We measured phage infectivity at transfer 4 and transfer 16. Previous studies with our study system observed signals of fluctuating selection dynamics in coevolution longer than 10 transfers [^3^](#_ENREF_3)^,^ [^4^](#_ENREF_4). The two timepoints, transfer 4 and 16, may very likely represent the arms race-like and fluctuating selection phases of coevolution, respectively. Phages were isolated from every microcosm at transfer 4 and 16. At each point in time, a reference pool of bacterial isolates was assembled as the challenge targets for phage infectivity measurement, as in previous studies [^4-6^](#_ENREF_4). Specifically, culture dilutions of every SL population, and one randomly chosen local population from every SS metapopulation, were spread onto LB agar plates, and one bacterial colony was chosen from each evolution line. This yielded a pool of 18 reference bacterial isolates at each timepoint.

We measured population-level phage infectivity, that is, infectivity of whole phage extracts from cultures, but not single phage isolates. For each SL population, the infectivity of a single phage sample was measured. For each SS metapopulation, we measured local population-level infectivity (five measures, each for one local population) as well as metapopulation-level infectivity (one measure for a mixture of five local populations). The reference bacterial isolates were separately grown in LB liquid medium on 96-well microplates for 24 h. For each phage sample, a line of phage extract (20 µL) was used to draw a line on an agar plate. After the phage line drying, we streaked culture of each reference bacterial isolates, and the ancestral bacterial strain, across the phage lines. After 24 h of incubation, we checked the growth of the bacterial isolates. Inhibition of bacterial growth indicated phage infectivity. Infectivity range of each phage sample was defined as the proportion of susceptible bacterial isolates among the total of 18 (Fig. S2). A phage population was considered as extinct if the phage extract did not inhibit the growth of the ancestral bacterial strain or any of the 18 reference bacterial isolates. We also measured the infectivity of the ancestral phage strain against the reference bacterial isolates, which was zero for both transfer 4 and transfer 16.

For each SS metapopulation, the difference of metapopulation-level infectivity from the maximum local population-level infectivity measure was calculated, termed as an “over-infectivity” index (Fig. S2). Positive values of this index indicate complementarity in infectivity profiles among the local populations. We did not expect negative over-infectivity values, as the metapopulation phage sample contained the most infective local sample. In practice, negative values may be observed as mixing five local phage samples led to dilution of the most infective local phage sample.

**Data analysis**

Data analyses were carried out in the R environment [^7^](#_ENREF_7). Comparison among habitat types in infectivity range was performed using two-sample Wilcoxon tests; and *P* values were adjusted using the Benjamini-Hochberg method. Departure of the over-infectivity index from the expected value of zero was analyzed using one-sample *t* test or one-sample Wilcoxon test, depending on the normality of error distribution.

**Supplementary references**

1 Bailey MJ, Lilley AK, Thompson IP, Rainey PB, Ellis RJ. Site directed chromosomal marking of a fluorescent pseudomonad isolated from the phytosphere of sugar beet; Stability and potential for marker gene transfer. Molecular Ecology. 1995;4(6):755-63.

2 Buckling A, Rainey PB. Antagonistic coevolution between a bacterium and a bacteriophage. Proc R Soc B. 2002;269(1494):931-6.

3 Hall AR, Scanlan PD, Morgan AD, Buckling A. Host-parasite coevolutionary arms races give way to fluctuating selection. Ecol Lett. 2011;14(7):635-42.

4 Lopez-Pascua L, Hall AR, Best A, Morgan AD, Boots M, Buckling A. Higher resources decrease fluctuating selection during host–parasite coevolution. Ecol Lett. 2014;17(11):1380-8.

5 Hall AR, Scanlan PD, Buckling A. Bacteria-phage coevolution and the emergence of generalist pathogens. The American Naturalist. 2011;177(1):44-53.

6 Zhang Q-G, Buckling A. Resource-dependent antagonistic coevolution leads to a new paradox of enrichment. Ecology. 2016;97(5):1319-28.

7 R Core Team. R: A language and environment for statistical computing. R Foundation for Statistical Computing. Vienna, Austria. 2024.

**Figure S1** A graphical illustration of our hypothesis. Fluctuating selection dynamics emerges when phage infectivity alleles match host resistance alleles, that is, each phage genotype infects specific corresponding host genotypes (different genotypes indicated by different line types). Allele frequency oscillates: at any given time, selection favours rare host alleles, which are less prone to infection by the prevailing parasite genotypes. Divergence in allele frequency dynamics would occur among local populations; and distinct combinations of bacterial and phage genotypes may be present in different local habitats. Thus the infectivity of a phage metapopulation would be contributed to by multiple phage strains with complementary infectivity profiles; and phages from several small habitats should have greater total infectivity ranges compared with single large habitats. In arms race-like coevolution, bacteria and phages are under directional selection to exceed the trait of the coevolutionary partner; and the infectivity range of a phage (meta)population is determined by the single, most infective, phage genotype. Single large habitats allow for more rapid emergence of generalist phage strains with broader infectivity ranges.


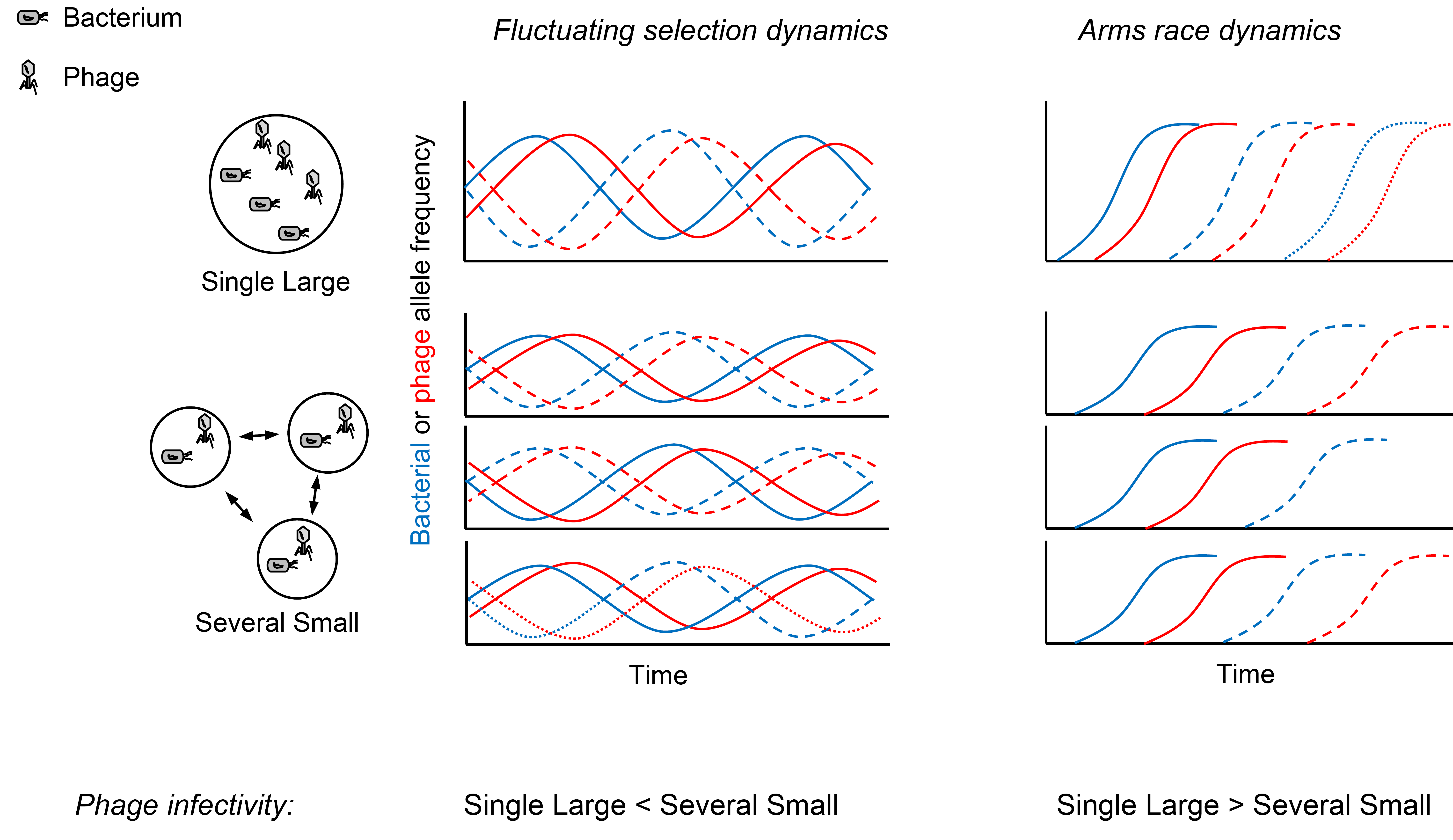


**Figure S2** A schematic illustration of the experimental design. The three types of evolution lines, single large (SL), several small (SS) with bacteria/phage dispersal and SS with phage dispersal, evolved for 16 transfers. Bacteria/phage dispersal within an SS metapopulations was carried out by supplementing each local populations with a mixture of samples from all the five local populations. For phage-only dispersal, only phage extracts were mixed and dispersed among local populations. To measure phage infectivity, one bacterial isolate was chosen from each evolution line, to assemble a reference bacterial pool. Susceptibility of the reference bacteria was tested against each phage population. For SS metapopulations, infectivity of each local population and that of the metapopulation (mixed sample of local populations) were both measured.


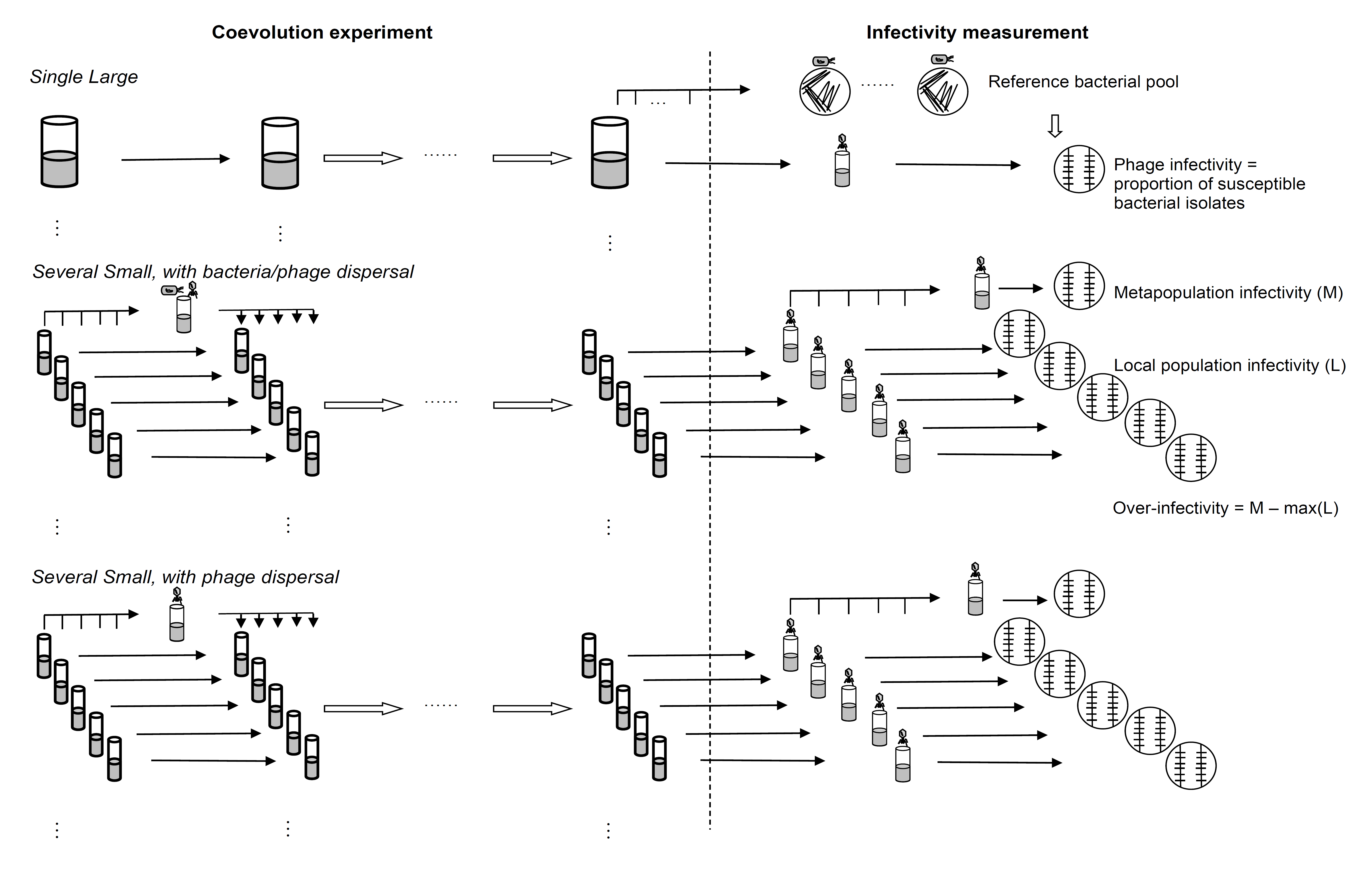


**Figure S3** Infectivity ranges of phage populations from single large habitats or metapopulations of several small habitats (A), and an over-infectivity index calculated for the two types of metapopulations of several small habitats (B), at an early point in time (transfer 4). In (A), habitats annotated with a same letter showed no significant difference (based on two-sample Wilcoxon tests, *P*_adj_ > 0.05; see details in Table S2). In (B), the difference of mean values from expected value of zero was indicated by annotated symbols, with asterisk and the text “ns” indicating significant and non-significant differences, respectively (based on one-sample *t* test or one-sample Wilcoxon test; see details in Table S3).


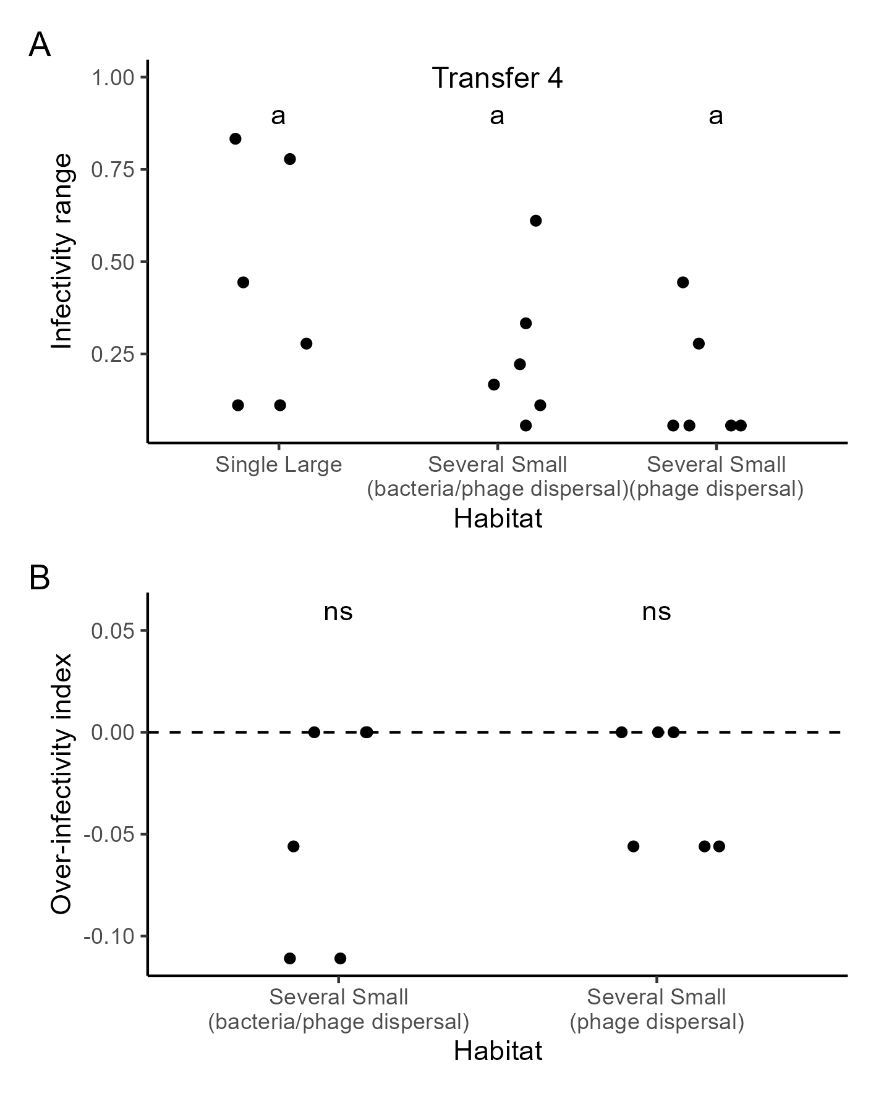


**Figure S4** The centrifuge tubes used to set up microcosms. Both types of tubes were U-bottomed; and inner diameters of the 10- and 100-mL tubes were 16 and 37 mm, respectively.


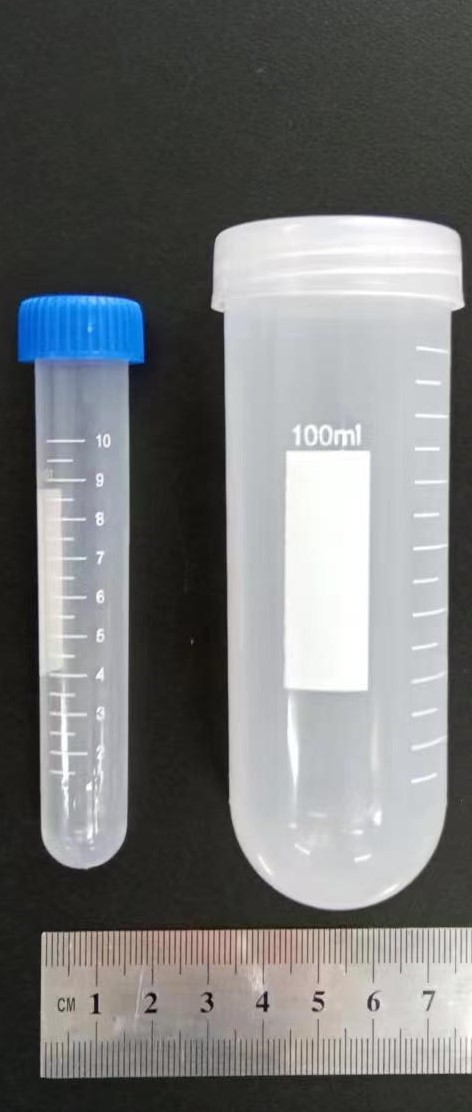


**Table S1** Summary of statistical analysis for departure of evolved phages’ infectivity from the ancestral phage infectivity (which was zero when tested against reference bacterial pools at both transfer 4 and transfer 16). Analysis was based on one-sample *t* test (for samples with normal distribution) or one-sample Wilcoxon test (for samples that did not show a normal distribution). “SL” represents “single large”; “SSA”, “several small” habitats with bacteria/phage dispersal; “SSB”, “several small” habitats with phage dispersal.

|  | Shapiro-Wilk normality test | One-sample *t* test | One-sample Wilcoxon test |
| --- | --- | --- | --- |
| *Transfer 4*  SL  SSA  SSB | *W* = 0.870, *P* = 0.225  *W* = 0.891, *P* = 0.322  *W* = 0.705, *P* = 0.007 | *t* = 3.27, *df* = 5, *P* = 0.022  *t* = 3.05, *df* = 5, *P* = 0.028 | *V* = 21, *P* = 0.031 |
| *Transfer 16*  SL  SSA  SSB | *W* = 0.894, *P* = 0.379  *W* = 0.552, *P* < 0.001  *W* = 0.640, *P* = 0.001 | *t* = 49.53, *df* = 5, *P* < 0.001 | *V* = 15, *P* = 0.048  *V* = 21, *P* = 0.031 |

**Table S2** Summary of statistical analysis of phage infectivity among habitat types, based on two-sample Wilcoxon tests. The Benjamini-Hochberg method was used for *P* value adjusting. “SL” represents “single large”; “SSA”, “several small” habitats with bacteria/phage dispersal; “SSB”, “several small” habitats with phage dispersal.

|  | Wilcoxon tests | Adjusted *P* values (HM ) |
| --- | --- | --- |
| *Transfer 4*  SL versus SSA  SL versus SSB  SSA versus SSB | *W* = 24, *P* = 0.375  *W* = 30, *P* = 0.060  *W* = 25, *P* = 0.281 | *P*_adjust_ = 0.375  *P*_adjust_ = 0.180  *P*_adjust_ = 0.375 |
| *Transfer 16*  SL versus SSA  SL versus SSB  SSA versus SSB | *W* = 11, *P* = 0.823  *W* = 2, *P* = 0.0161  *W* = 0, *P* = 0.005 | *P*_adjust_ = 0.823  *P*_adjust_ = 0.024  *P*_adjust_ = 0.015 |

**Table S3** Summary of statistical analysis for departure of the over-infectivity index from zero, based on one-sample *t* test (for samples with normal distribution) or one-sample Wilcoxon test (for samples that did not show a normal distribution). “SSA”, “several small” habitats with bacteria/phage dispersal; “SSB”, “several small” habitats with phage dispersal.

|  | Shapiro-Wilk normality test | One-sample *t* test | One-sample Wilcoxon test |
| --- | --- | --- | --- |
| *Transfer 4*  SSA  SSB | *W* = 0.775, *P* = 0.035  *W* = 0.683, *P* = 0.004 |  | *V* = 0, *P* = 0.346  *V* = 10, *P* = 0.098 |
| *Transfer 16*  SSA  SSB | *W* = 0.684, *P* = 0.006  *W* = 0.861, *P* = 0.193 | *t* = 2.667, *df* = 5, *P* = 0.045 | *V* = 0, *P* = 0.346 |
